# Synthesis, Properties, and Biodegradability of Novel Sequence-Controlled Copolyesters Composed of Glycolic Acid, Dicarboxylic Acids, and C_3_ or C_4_ Diols

**DOI:** 10.3390/polym15051155

**Published:** 2023-02-24

**Authors:** Yuushou Nakayama, Keitaro Fukumoto, Yuji Kusu, Ryo Tanaka, Takeshi Shiono, Norioki Kawasaki, Naoko Yamano, Atsuyoshi Nakayama

**Affiliations:** 1 Department of Applied Chemistry, Graduate School of Advanced Science and Engineering, Hiroshima University, 1-4-1 Kagamiyama, Higashi-Hiroshima 739-8527, Hiroshima, Japan; 2 Biomedical Research Institute, National Institute of Advanced Industrial Science and Technology (AIST), 1-8-31 Midorigaoka, Ikeda 563-8577, Osaka, Japan

**Keywords:** sequence-controlled copolyesters, alkanediols, glycolic acid, dicarboxylic acid, biodegradation in seawater, hydrolysis

## Abstract

We have previously reported that sequence-controlled copolyesters such as poly((ethylene diglycolate) terephthalate) (poly(GEGT)) showed higher melting temperatures than those of the corresponding random copolymers and high biodegradability in seawater. In this study, to elucidate the effect of the diol component on their properties, a series of new sequence-controlled copolyesters composed of glycolic acid, 1,4-butanediol or 1,3-propanediol, and dicarboxylic acid units was studied. 1,4-Butylene diglycolate (GBG) and 1,3-trimethylene diglycolate (GPG) were prepared by the reactions of 1,4-dibromobutane or 1,3-dibromopropane with potassium glycolate, respectively. Polycondensation of GBG or GPG with various dicarboxylic acid chlorides produced a series of copolyesters. Terephthalic acid, 2,5-furandicarboxylic acid, and adipic acid were used as the dicarboxylic acid units. Among the copolyesters bearing terephthalate or 2,5-furandicarboxylate units, the melting temperatures (*T*_m_) of the copolyesters containing 1,4-butanediol or 1,2-ethanediol units were substantially higher than those of the copolyester containing the 1,3-propanediol unit. Poly((1,4-butylene diglycolate) 2,5-furandicarboxylate) (poly(GBGF)) showed a *T*_m_ at 90 °C, while the corresponding random copolymer was reported to be amorphous. The glass-transition temperatures of the copolyesters decreased as the carbon number of the diol component increased. Poly(GBGF) was found to show higher biodegradability in seawater than that of poly(butylene 2,5-furandicarboxylate) (PBF). On the other hand, the hydrolysis of poly(GBGF) was suppressed in comparison with that of poly(glycolic acid). Thus, these sequence-controlled copolyesters have both improved biodegradability compared to PBF and lower hydrolyzability than PGA.

## 1. Introduction

Because of the problems of global warming and plastic pollution, bioplastics including bio-based polymers and/or biodegradable polymers are attracting current attention. Aliphatic polyesters such as poly(L-lactic acid) [1,2,3] and poly(glycolic acid) (PGA) [3,4] are known as representative biodegradable polymers. On the other hand, aromatic polyesters such as poly(ethylene terephthalate) (PET), poly(trimethylene terephthalate) (PTT), and poly(butylene terephthalate) (PBT) have excellent thermal and mechanical properties and are widely used as commodity plastics as well as fibers. However, aromatic polyesters generally have poor biodegradability, which leads to the accumulation of waste plastics in the environment. From these backgrounds, aliphatic–aromatic copolyesters have been studied to develop polymers with both good physical properties and biodegradability [5,6]. The production of some aliphatic– aromatic copolyesters such as poly(butylene terephthalate-*co*-adipate) (PBAT) [7] has been commercialized. 

Some copolyesters containing lactic acid have been reported. Olewnik [8] and Gara [9] synthesized poly(ethylene terephthalate-*co*-lactic acid) from the reaction of bis(2-hydroxyethyl terephthalate) (BHET) with l-lactic acid oligomers and that of BHET with lactide, respectively. The content of aromatic ester units in the copolymers affected the melting temperature (*T*_m_) of the copolymer, and the copolymer with about 80% aromatic ester units showed a *T*_m_ of 215 °C, while the copolymer with 60% aromatic ester units showed no *T*_m_. PGA is a highly biodegradable and hydrolyzable polymer [3,4], and the incorporation of glycolic acid components into other polymers has also been studied. Olewnik et al. reported the synthesis of poly(ethylene terephthalate-*co*-glycolic acid) from the reaction of BHET with glycolic acid oligomers [10]. The *T*_m_ of the copolyester with 36 mol% of glycolic acid was detected at 166 °C. Ding et al. and Tial et al. recently independently reported poly(butylene succinate-*co*-glycolic acid) (PBSGA), which showed higher enzymatic hydrolyzability than poly(butylene succinate) [11,12]. The structures of these copolymers are random or block-like.

We recently synthesized ABAC-type sequence-regulated aliphatic–aromatic copolyesters composed of glycolic acid, ethylene glycol, and terephthalic acid units [13]. In this study, poly((ethylene diglycolate) terephthalate) (poly(GEGT)) was found to show relatively high *T*_m_ in comparison with the corresponding random copolymer. Poly(GEGT) also exhibited high biodegradability in seawater. 

It is known that the properties of polyesters significantly depend on whether the carbon numbers of the components are odd or even, where the polyesters composed of diols with even carbon numbers tend to exhibit higher *T*_m_ than those with odd carbon numbers [14,15,16]. 

To elucidate the effect of the diol structure on the properties of the ABAC-type sequence-controlled aliphatic–aromatic copolyesters, in this study, a series of the sequence-controlled copolyesters composed of glycolic acid, dicarboxylic acids, and 1,4-butanediol or 1,3-propanediol was synthesized. Their thermal properties, biodegradability in seawater, and hydrolyzability were investigated. 

## 2. Materials and Methods

### 2.1. General

All polymerization reactions were conducted under a nitrogen (N_2_) atmosphere using Schlenk techniques. ^1^H and ^13^C NMR spectra were recorded on Varian system 500 (Santa Clara, CA, USA) or JEOL Lambda 500 spectrometers (Tokyo, Japan) (500 MHz for ^1^H nucleus or 125 MHz for ^13^C nucleus, respectively). Chemical shifts of ^1^H NMR spectra were calibrated by using the signal for the residual protons in chloroform-d (δ = 7.26 ppm) or in 1,1,2,2-tetrachloroethane (δ = 6.00 ppm). Chemical shifts of ^13^C NMR spectra were calibrated by using the signal for the carbons in chloroform-d (δ = 77.16 ppm) or in 1,1,2,2-tetrachloroethane (δ = 74.0 ppm). Electrospray ionization mass spectrometry (ESI-MS) experiments were carried out on a Thermo Fisher Scientific LTQ Orbitrap XL (Waltham, MA, USA) at the Natural Science Center for Basic Research and Development (N-BARD), Hiroshima University. Spectra were recorded in a positive ion mode using a spray voltage of 5.0 kV and mass range of *m*/*z* 100–2000. Samples were dissolved in methanol as a matrix solution. Elemental analysis was performed on a Perkin Elmer 2400 series II CHN/O analyzer (Waltham, MA, USA). Molecular weights and polydispersities of the polymers soluble in tetrahydrofuran (THF) were determined by gel permeation chromatography (GPC) measurements on a Tosoh GPC system (HLC-8320, Tokyo, Japan) equipped with an RI detector connected to a series of TSK gel columns G2000, G3000, G4000, and G5000. THF was used as an eluent at a flow rate of 1.0 mL/ min at 40 °C. Molecular weights and polydispersities of the polymers soluble in CHCl_3_ but insoluble in THF were determined by GPC measurements on a JASCO GPC system (PU-2080, Tokyo, Japan) equipped with an RI detector. CHCl_3_ was used as an eluent at a flow rate of 1.0 mL/min at 40 °C. The GPC curves were calibrated using standard polystyrenes. Molecular weights and polydispersities of the polymers insoluble in both THF and chloroform were determined using a GPC system (HLC-8220, TOSOH Co., Tokyo, Japan). Poly(methyl methacrylate) was used as a standard substance. A column (TSK gel Super HM-N, TOSOH Co., Tokyo, Japan) was used with 1,1,1,3,3,3-hexafluoro-2-propanol (HFIP) (Central Glass Co. Ltd., Tokyo, Japan) as an eluent (0.2 mL/min, 40 °C). The melting temperature ™, melting enthalpy (Δ*H*_m_), and glass transition temperature (*T*_g_) of the polymers were measured by differential scanning calorimetry (DSC) using a Seiko DSC 6220 apparatus (Chiba, Japan). The heating rate was 10 °C/ min in a nitrogen stream. The *T*_m_ and Δ*H*_m_ were read from the first heating scan. The *T*_g_ values of the polymers were defined as the midpoint temperature of a heat capacity change in the second heating scan. 

### 2.2. Materials

Glycolic acid (GA), thionyl chloride (SOCl_2_), 1,4-butanediol (BD), adipic acid (AA), 2,5-furandicarboxylic acid (FDCA), and *N*,*N*-dimethylacetamide (DMAc) were purchased from Tokyo Chemical Industry Co. Potassium hydrogen carbonate and *o*-dichlorobenzene (ODCB) were purchased from Wako Pure Chemical Industries, Ltd. *N*,*N*-Dimethylformamide (DMF), 1,1,2,2-tetrachloroethane (TCE), pyridine, acetone, chloroform, methanol, THF, dimethyl sulfoxide (DMSO), and sulfuric acid (H_2_SO_4_) were purchased from Kanto Chemical Co., Inc (Tokyo Japan). 1,3-Dibromopropane (DBP), terephthaloyl dichloride (TC), and adipoyl dichloride (AC) were purchased from Tokyo Chemical Industry Co. (Tokyo Japan). 1,4-Dibromobutane, and poly(glycolic acid) (PGA) were purchased from Sigma-Aldrich, Inc. (St. Louis, MO, USA) Poly(butylene furanoate) (PBF) [17] and poly(butylene adipate) (PBA) [18] were prepared according to the literatures. 2,5-Furandicarbonyl dichloride (FC) was prepared from the reaction of FDCA with SOCl_2_ according to the literature [19]. 

### 2.3. Synthesis of 1,4-Butylene Diglycolate (GBG)

First, potassium glycolate was prepared by the reaction of glycolic acid (0.134 mol) and potassium hydrogen carbonate (0.160 mol) in water (40 mL) at r.t. for 1 h, followed by evaporation to dryness. 1,4-Dibromobutane (0.016 mol) was added to a solution of potassium glycolate (0.035 mol) in DMF (30 mL) and the mixture was stirred at 80 °C for 2 h. Then, DMF was removed from the resulting mixture under reduced pressure to yield a solid. Acetone was added to the solid and the mixture was filtrated through silica gel. The filtrate was dried in air to give GBG as a white solid in 67% yield. ^1^H NMR (500 MHz, CDCl_3_, r.t.): δ 4.23 (t, 4H, *J* = 6.0 Hz, OC*H*_2_CH_2_CH_2_C*H*_2_O), 4.17 (s, 4H, COC*H*_2_OH), 2.53 (br, 2H, COCH_2_O*H*), 1.75 (m, 4H, OCH_2_C*H*_2_C*H*_2_CH_2_O). ^13^C NMR (125 MHz, CDCl_3_, r.t.): δ 173.50 (*C*OCH_2_OH), 64.90 (CO*C*H_2_OH), 60.65 (O*C*H_2_CH_2_CH_2_*C*H_2_O), 25.18 (OCH_2_*C*H_2_*C*H_2_CH_2_O). ESI-MS calculated for C_8_H_14_O_6_Na^+^: 229.06881, observed: 229.0684.

### 2.4. Synthesis of 1,3-Propylene Diglycolate (GPG)

First, potassium glycolate was prepared by the reaction of glycolic acid (0.134 mol) and potassium hydrogen carbonate (0.160 mol) in water (40 mL) at r.t. for 1 h, followed by evaporation to dryness. 1,3-Dibromopropane (0.008 mol) was added to a solution of potassium glycolate (0.018 mol) in DMF (25 mL) and the mixture was stirred at 80 °C for 2 h. Then, DMF was removed from the resulting mixture under reduced pressure to yield a solid. The solid was purified by column chromatography (silica gel) using acetone as an eluent and recrystallized from acetone to give GPG as a white solid in 40% yield. ^1^H NMR (500 MHz, CDCl_3_, r.t.) δ 4.26 (t, 4H, *J* = 6.3 Hz, OC*H_2_*CH_2_C*H_2_*O), 4.17 (s, 4H, COC*H*_2_OH), 2.32 (br, 2H, O*H*), 2.03 (quin, 2H, *J* = 6.3 Hz, CH_2_C*H_2_*CH_2_). ^13^C NMR (125 MHz, CDCl_3_) δ 173,4 (*C*OCH_2_OH), 61.9 (O*C*H_2_CH_2_*C*H_2_O), 60.7 (CO*C*H_2_OH), 27.9 (OCH_2_*C*H_2_CH_2_O). ESI-MS calculated for C_7_H_12_O_6_Na: 215.05261, observed: 215.05231. Anal. Calcd for C_7_H_12_O_6_: C, 43.75; H, 6.29. Found: C, 43.88, H, 6.29. 

### 2.5. Polycondensation of GBG and TC

A typical procedure: A Schlenk tube was charged with GBG (2.13 mmol), TC (2.13 mmol), and 1,1,2,2-tetrachloroethane (2.0 mL). Then, pyridine (2.0 mL) was added to the Schlenk tube at 0 °C and the mixture was stirred for 10 min, followed by further stirring at room temperature for 2 h to promote the polycondensation. The resulting mixture was poured into excess methanol. The precipitated polymer was collected by centrifugation and dried in vacuo to give poly(GBGT) as a white solid in 86% yield. ^1^H NMR (500 MHz, CD_2_Cl_4_, 130 °C): δ 8.19 (s, 4H, COC_6_*H*_4_CO), 4.89 (s, 4H, COC*H*_2_O), 4.23 (brs, 4H, OC*H*_2_CH_2_CH_2_C*H*_2_O), 1.75 (brs, 4H, OCH_2_C*H*_2_C*H*_2_CH_2_O). ^13^C NMR (125 MHz, CD_2_Cl_4_, 130 °C): δ 167.2 (*C*OCH_2_O), 165.0 (*C*OC_6_H_4_*C*O), 133.8 (*ipso*-*C*_6_H_4_), 129.8 (other *C*_6_H_4_), 64.8 (CO*C*H_2_O), 61.6 (O*C*H_2_CH_2_CH_2_*C*H_2_O), 25.3 (OCH_2_*C*H_2_*C*H_2_CH_2_O). 

### 2.6. Polycondensation of GPG and TC

A Schlenk tube was charged with GPG (2.13 mmol), TC (2.13 mmol), and 1,1,2,2-tetrachloroethane (2.0 mL). Then, pyridine (2.0 mL) was added to the Schlenk tube at 0 °C and the mixture was stirred for 10 min, followed by further stirring at room temperature for 2 h to promote the polycondensation. The resulting mixture was poured into excess methanol. The precipitated polymer was collected by centrifugation and dried in vacuo to give poly(GPGT) as a white solid in 64% yield. ^1^H NMR (500 MHz, CD_2_Cl_4_, 130 °C): δ 8.17 (s, 4H, COC_6_*H*_4_CO), 4.89 (s, 4H, COC*H*_2_O), 4.28 (t, 4H, OC*H*_2_CH_2_C*H*_2_O), 2.04 (quin, 2H, OCH_2_C*H*_2_CH_2_O).

### 2.7. Polycondensation of GBG and FC

A typical procedure: A Schlenk tube was charged with GBG (1.05 mmol) and FC (1.16 mmol). Then, pyridine (1.5 mL) was added to the Schlenk tube at 0 °C and the mixture was stirred for 10 min, followed by further stirring at 70 °C for 2 h to promote the polycondensation. The resulting mixture was poured into excess methanol. The precipitated polymer was collected by centrifugation and dried in vacuo to give poly(GBGF) as a white solid in 92% yield. ^1^H NMR (500 MHz, CD_2_Cl_4_, 130 °C): δ 7.34 (s, 4H, COC_4_*H*_4_OCO), 4.85 (s, 4H, COC*H*_2_O), 4.20 (brs, 4H, OC*H*_2_CH_2_CH_2_C*H*_2_O), 1.73 (brs, 4H, OCH_2_C*H*_2_C*H*_2_CH_2_O). 

### 2.8. Polycondensation of GPG and FC

A typical procedure: A Schlenk tube was charged with GPG (0.35 mmol) and FC (0.39 mmol). Then, pyridine (0.5 mL) was added to the Schlenk tube at 0 °C for 10 min, followed by further stirring at r.t. for 2 h to promote the polycondensation. The resulting mixture was poured into excess methanol. The precipitated polymer was collected by centrifugation and dried in vacuo to give poly(GPGF) as a light brown solid in 94% yield. ^1^H NMR (500 MHz, CD_2_Cl_4_, 130 °C): δ 7.34 (s, 4H, COC_4_*H*_4_OCO), 4.86 (s, 4H, COC*H*_2_O), 4.26 (t, 4H, OC*H*_2_CH_2_C*H*_2_O), 2.04 (quin, 2H, OCH_2_C*H*_2_CH_2_O).

### 2.9. Polycondensation of GBG and AC

A typical procedure: A Schlenk tube was charged with GBG (1.71 mmol), AC (1.71 mmol), and 1,1,2,2-tetrachloroethane (1.5 mL). Then, pyridine (1.0 mL) was added to the Schlenk tube at 0 °C and the mixture was stirred for 10 min, followed by further stirring at 50 °C for 3.5 h to promote the polycondensation. The resulting mixture was poured into excess methanol. The precipitated polymer was collected by centrifugation and dried in vacuo to give poly(GBGA) as a white solid in 92% yield. ^1^H NMR (500 MHz, CD_2_Cl_4_, 130 °C): δ 4.60 (s, 4H, COC*H*_2_O), 4.19 (t, 4H, OC*H*_2_CH_2_CH_2_C*H*_2_O), 2.47 (t, 4H, COC*H*_2_CH_2_CH_2_C*H*_2_CO), 1.73 (m, 8H, OCH_2_C*H*_2_C*H*_2_CH_2_O and COCH_2_C*H*_2_C*H*_2_CH_2_CO).

### 2.10. Biodegradation of the Polymers with Seawater Monitored by Biochemical Oxygen Demand (BOD) Method

Biodegradation lab tests of the polymers in seawater were evaluated from the determination of oxygen consumption using a BOD tester (TAITEC, BOD200F, Saitama, Japan). Seawater was taken at the shoreline from the sea surface of Osaka South Port area with a bucket. The seawater was used within one or two days. Typically, 30 mg of polymer specimen was added into each 250 mL BOD testing bottle and then 200 mL supernatant of seawater was added. Evolved carbon dioxide (CO_2_) was removed using calcium hydroxide from the BOD closed system. The biodegradation tests were carried out at 27 °C with stirring for 28 days. The observed O_2_ consumption volume was corrected by subtraction of the O_2_ consumption volume of the control. The theoretical O_2_ consumption volume was calculated according to the structural formula of each polymer assuming that degraded products are completely mineralized to CO_2_. Biodegradation (%) of each polymer was calculated according to the following equation:

% Biodegradation = (Observed O_2_ consumption volume/theoretical O_2_ consumption volume) × 100

Each degradation test was repeated twice and averaged. 

### 2.11. Hydrolysis of the Polymers Monitored by TOC Method

A polymer sample (10 mg) was added to a vial filled with 2.5 mL H_2_O. The reactions were carried out by shaking the vial at 45 °C for 1, 3, and 7 days separately. Finally, the solution was filtered and stored in the refrigerator (−30 °C) until the total organic carbon concentration (TOC) measurement. The weight loss was evaluated by using a TOC analyzer (Shimadzu TOC-VCSH, Kyoto, Japan). 

## 3. Results and Discussion

### 3.1. Synthesis of the Polymers

#### 3.1.1. Preparation of GBG and GPG

**Scheme 1:**
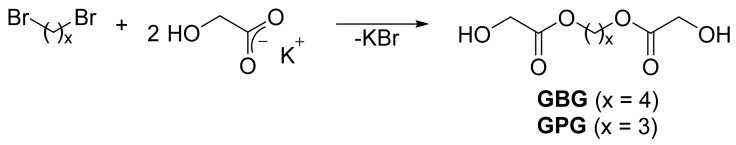
Synthesis of GBG and GPG.

**Figure 1:**
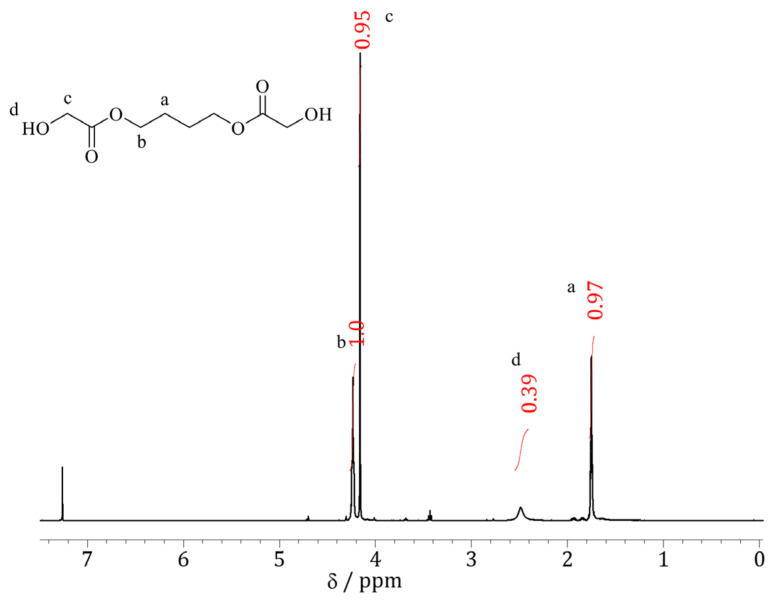
^1^H NMR spectrum of GBG (CDCl_3_, 500 MHz, r.t.). Red color: relative intensity.

1,4-Butylene diglycolate (GBG) and 1,3-propylene diglycolate (GPG) were synthesized by the reaction of 1,4-dibromobutane or 1,3-dibromopentane with potassium glycolate in *N*,*N*-dimethylformamide (DMF), similarly to GEG (Scheme 1) [13]. After the reaction of 1,4-dibromobutane or 1,3-dibromopentane with potassium glycolate, the acetone solution of the crude product was filtrated through silica gel followed by evaporation to give GBG in 67% yield. The ^1^H NMR spectrum of GBG is shown in Figure 1. GPG was purified by silica gel column chromatography and recrystallization and obtained in 40% yield (Figure S1). 

#### 3.1.2. Synthesis of Poly(GBGT) and Poly(GPGT)

**Scheme 2:**
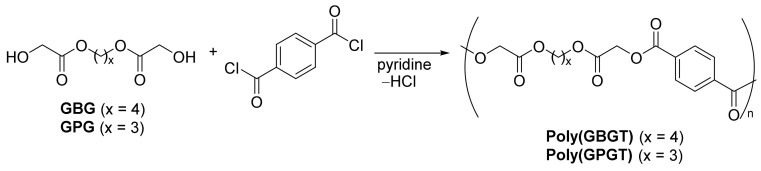
Synthesis of poly(GBGT) and poly(GPGT).

**Figure 2:**
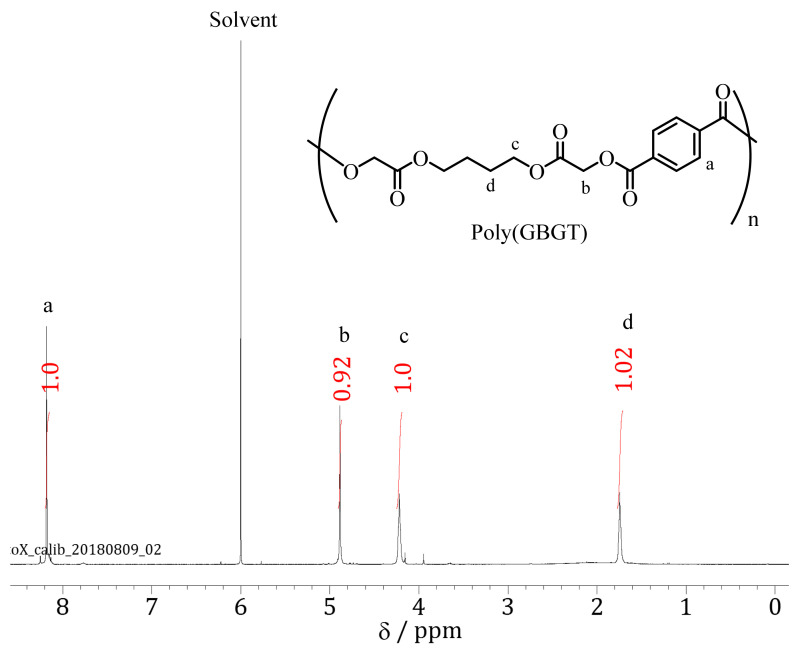
^1^H NMR spectrum of poly(GBGT) (Table 1, Run 1) (C_2_D_2_Cl_4_, 500 MHz, 130 °C). Red color: relative intensity.

**Figure 3:**
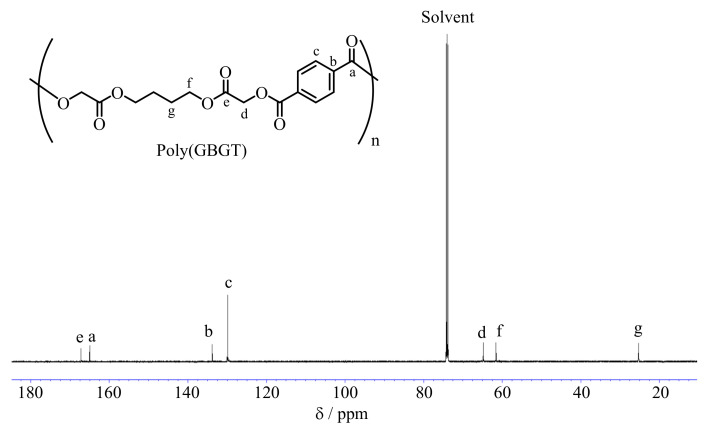
^13^C NMR spectrum of poly(GBGT) (Table 1, Run 1) (C_2_D_2_Cl_4_, 125 MHz, 130 °C).

Similarly to the synthesis of poly(GEGT) [13], the polycondensation of GBG or GPG with terephthaloyl dichloride (TC) gave the corresponding sequence-controlled copolyesters, poly(GBGT) and poly(GPGT), respectively (Scheme 2, Table 1). The obtained poly(GBGT)s were hardly soluble in common organic solvents such as tetrahydrofuran (THF) and chloroform, so their GPC analysis was performed using 1,1,1,3,3,3-hexafluoro-2-propanol as an eluent to indicate their rather low molecular weight (*M*_n_ ≤ 2,400). The attempted polycondensations of GBG with TC under various conditions such as different feed ratios of GBG or GPG against TC, different solvents (1,1,2,2-tetrachloroethane (TCE, DMF, dimethyl acetamide (DMAc), and dimethyl sulfoxide (DMSO)) and different temperatures did not enhance the molecular weight of the resulting poly(GBGT). The obtained poly(GPGT) (Figure S2) had a slightly higher molecular weight than that of poly(GBGT) and was also insoluble in THF but soluble in CHCl_3_. The lower polymer yield of poly(GPGT) than that of poly(GBGT) should come from the higher solubility of poly(GPGT) in the precipitation solvent, MeOH. This could be attributed to the lower crystallinity of poly(GPGT) than that of poly(GBGT) (vide infra). The ^1^H and ^13^C NMR spectra of poly(GBGT) were recorded in TCE-d_2_ at 130 °C as shown in Figure 2 and Figure 3, respectively. The spectra showed four (^1^H NMR) and seven (^13^C NMR) sharp signals, respectively, indicating the regular comonomer sequence of the copolymer as expected. 

#### 3.1.3. Synthesis of Poly(GBGF) and Poly(GPGF)

**Scheme 3:**
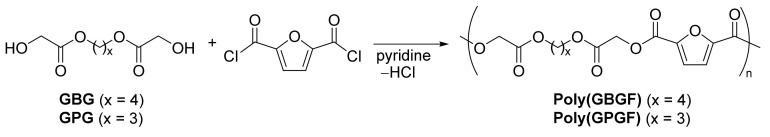
Synthesis of poly(GBGF) and poly(GPGF).

2,5-Furandicarboxylic acid (FDCA) is currently attracting attention as a renewable bio-based chemical [20,21]. Poly(ethylene furanoate) (PEF) [22,23], poly(1,3-propylene furanoate) (PPF) [24,25], and poly(butylene furanoate) (PBF) [17] made from FDCA and ethylene glycol, 1,3-propanediol, or 1,4-butanediol, respectively, are potential alternatives to PET, PTT, and PBF having high gas-barrier properties [17,23]. Similar to the synthesis of poly(GBGT) and poly(GPGT), the polycondensation of GBG or GPG with 2,5-furandicarbonyl dichloride (FC) also gave the corresponding sequence-controlled copolyesters, poly(GBGF) and poly(GPGF), respectively (Scheme 3, Table 2, Figures S3 and S4). The obtained poly(GBGF)s were soluble in CHCl_3_, and their GPC analyses were performed using CHCl_3_ as an eluent. A poly(GBGF) with relatively high molecular weight (*M*_n_ = 14 kg mol^−1^) was obtained from the polycondensation of GBG with FC at 70 °C in pyridine without additional solvent (run 7). The molecular weights of the obtained poly(GPGF)s were lower than those of poly(GBGF)s. The higher molecular weight poly(GPGF) (run 11, *M*_n_ = 10 kg mol^−1^) was obtained by re-precipitation of the lower molecular weight poly(GPGF) (run 10, *M*_n_ = 6 kg mol^−1^) in MeOH. 

#### 3.1.4. Synthesis of Poly(GBGA)

**Scheme 4:**
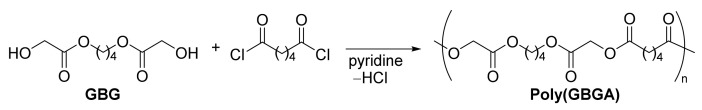
Synthesis of poly(GBGA).

Because aliphatic polyesters tend to show higher biodegradability than aromatic polyesters in general, the polycondensation reaction of GBG with adipoyl dichloride was also performed to produce poly(GBGA) using TCE as a solvent (Scheme 4, Table 3, Figure S5). The polymerization was conducted at r.t., 50 °C, and 70 °C, and produced poly(GBGA) with the highest molecular weight (*M*_n_) of 21 kg mol^−1^ in the highest yield at 50 °C. The polycondensation of GBG and adipoyl dichloride at 70 °C resulted in a much lower yield than that at 50 °C and gave an orange-colored polymer with lower molecular weight, suggesting some side reactions at 70 °C. 

### 3.2. Thermal Properties of the Polymers

The thermal properties of the obtained copolyesters were evaluated by DSC analysis, summarized in Table 4, which includes the data of the reported poly(GEGT), poly(GEGF), and poly(GEGA) [13] for comparison. 

Among the terephthalate polymers, poly(GBGT) showed *T*_m_ at 202 °C, which is similar to those of homopolymers PGA (around 220 °C [26,27]) and PBT (around 225 °C [28,29]). In general, random copolymers tend to show lower *T*_m_ than each homopolymer [10]. We believe that the regular sequence of poly(GBGT) should contribute to its relatively high *T*_m_. In contrast, the *T*_m_ of poly(GPGT) was observed at 121 °C, which is significantly lower than those of the poly(GBGT) and poly(GEGT) (209 °C) [13]. The Δ*H*_m_ value of the poly(GPGT) (12 J/g) was also lower than those of the poly(GBGT) (28 J/g) and poly(GEGT) (20 J/g). These results indicate that the crystallinity of the poly(GPGT) is lower than those of the poly(GBGT) and poly(GEGT). We speculate that these data suggest the odd–even effect of the diol carbon numbers (“x” in Scheme 1, Scheme 2, Scheme 3 and Scheme 4) on their crystallization, where the polymers with diols composed of even number carbon atoms tend to have higher *T*_m_ than odd ones [14,15]. It is notable that the poly(GBGT) showed a melting transition also in the second heating scan (Figure S6) in contrast to the poly(GEGT) [13] and poly(GPGT) (Figure S7) showing no melting transition in the second heating scan, indicating a faster crystallization rate of the poly(GBGT) than those of the poly(GEGT) and poly(GPGT). The *T*_g_ values of these polymers were lowered with an increased diol carbon number, where the *T*_g_ of poly(GBGT) (9 °C) was lower by 39 °C than that of poly(GEGT) (48 °C). 

The poly(GBGF), which contains 67 mol% of glycolate units, showed *T*_m_ at 93 °C (Figure S8). In sharp contrast, the reported random copolymer with a similar composition (glycolate-content = 60 mol%) did not show *T*_m_ [30,31]. Thus, the regular sequence of the poly(GBGF) enhanced its crystallinity. The *T*_m_ of the poly(GBGF) is lower than those of PGA, PBF (around 170 °C) [17,32,33], and poly(GEGF) (127 °C) [13]. In contrast to the poly(GBGF), poly(GEGF), and reported PPF (*T*_m_ = 170—180 °C) [24,25], the poly(GPGF) showed no melting transition, indicating its amorphous nature (Figure S9). These results should also suggest the odd–even effect of the diol carbon numbers (“x” in Scheme 1, Scheme 2, Scheme 3 and Scheme 4) on their crystallization behaviors. The *T*_g_ values of these polymers also decreased with increasing the diol carbon number, where the *T*_g_ of poly(GBGF) (33 °C) was lower by 35 °C than that of poly(GEGF) (68 °C). 

The poly(GBGA) showed *T*_m_ at 72 °C (Figure S10), similar to those of poly(GEGA) (67 and 77 °C) [4] and higher than that of PBA (around 57 °C) [34,35]. These results are in sharp contrast to the fact that the corresponding random copolymer of poly(butylene adipate-*co*-glycolic acid) with similar composition (glycolate-content = 60 mol%) was reported to have no crystallinity [36]. The *T*_g_ of poly(GBGA) (−31 °C) was also lower by 42 °C than that of poly(GEGA) (13 °C) [13].

### 3.3. Biodegradability of the Polymers

Marine pollution by microplastics is one of the most concerning environmental problems [37,38]. While it is important to prevent plastic runoff by recycling, the use of biodegradable polymers should be effective for applications where recycling is difficult. From this background, the polymers biodegradable in seawater are attracting current attention [39]. Poly(3-hydroxyalkanoate)s such as poly(3-hydroxybutyrate) (PHB), poly(3-hydroxybutyrate-*co*-3-hydroxyvalerate) (PHBV), and poly(3-hydroxybutyrate-*co*-3-hydroxyhexanoate) (PHBH) are known for their high biodegradability in seawater, showing almost complete degradation within several weeks [40,41]. 

**Figure 4:**
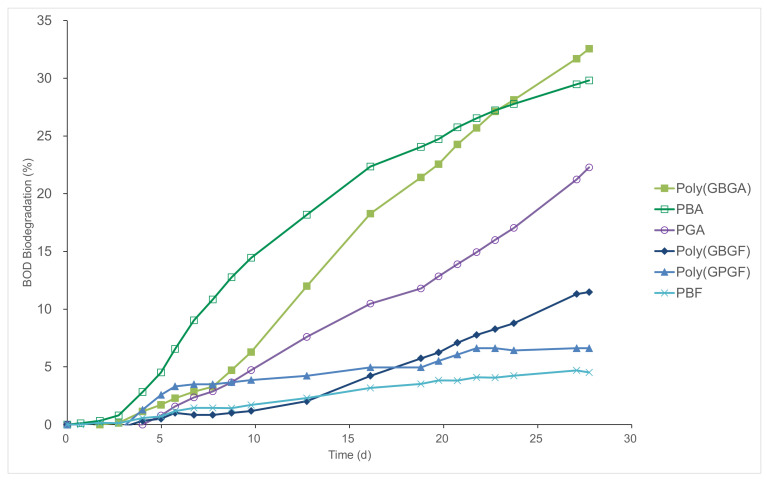
Biodegradation (BOD) of the homo- and copolyesters in seawater at 27 °C.

The biodegradation tests in seawater were performed using the samples of poly(GBGF) (run 5), poly(GPGF) (run 10), and poly(GBGA) (run 12) at 27 °C (Figure 4). For comparison, purchased PGA (*M*_n_ = 38 kg/mol, *M*_w_/*M*_n_ = 2.6) and separately synthesized PBF (*M*_n_ = 27 kg/mol, *M*_w_/*M*_n_ = 1.6) and PBA (*M*_n_ = 12 kg/mol, *M*_w_/*M*_n_ = 1.3) were also tested. Because of their very low molecular weights, poly(GBGT) and poly(GPGT) were not used in the biodegradation tests. The degradation degrees in these tests were evaluated by the biochemical oxygen demand (BOD) measurements. 

The poly(GBGF) and poly(GPGF) samples exhibited 11.5% and 6.5% biodegradation after 28 days, respectively, which are intermediate between those of PGA (22.3%) and PBF (4.5%). Therefore, the biodegradability of the poly(GBGF) and poly(GPGF) were improved in comparison with PBF by the introduction of glycolate units. The poly(GBGF) and poly(GPGF) have higher biodegradability than representative biodegradable copolyester, poly(butylene adipate-*co*-terephthalate) (PBAT), which was reported to show the BOD biodegradation of around 1% after 4 weeks in similar laboratory tests [41], although lower than that of poly(GEGT), which showed a biodegradation of 30% within 29 days [13]. The slow but certain biodegradation of the PBF homopolymer sample was observed, possibly due to its relatively low molecular weight. PBA was reported to show higher biodegradability in seawater than poly(butylene succinate) and poly(ethylene succinate) [40]. The poly(GBGA) sample showed a high biodegradation of 32.6% after 28 days similar to that of PBA (29.8%). The slower biodegradation of the PGA than those of the PBA and poly(GBGA) samples could come from the higher molecular weight of the used PGA sample. 

**Figure 5:**
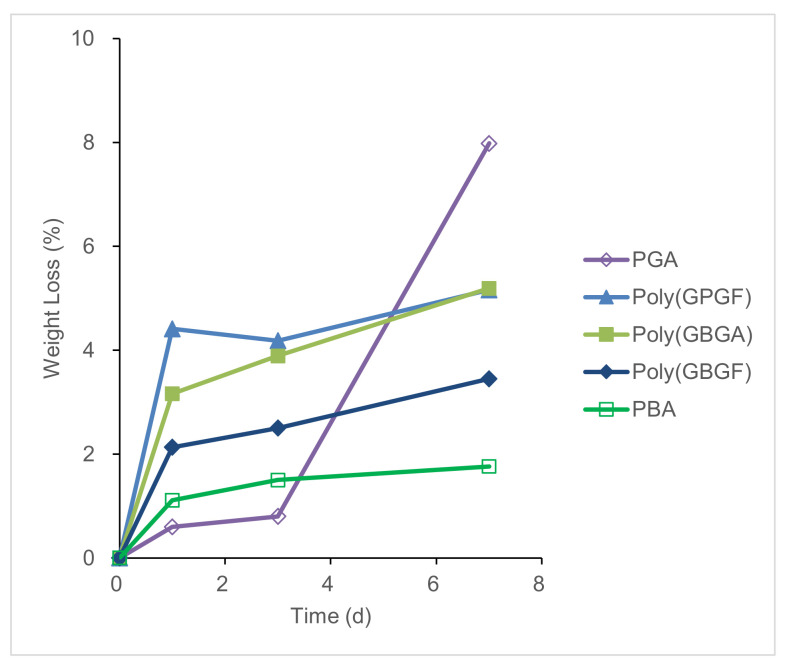
Hydrolytic degradation of the homo- and copolyesters at 45 °C.

PGA has high hydrolyzability, which leads to its high biodegradability and also to low stability in ambient conditions to limit its applications [3,4]. Figure 5 shows the results of the hydrolysis tests of these polymers at 45 °C. The degradation degrees in these tests were determined from the total organic carbon (TOC) measurement of the supernatant solution. The weight loss on the first day is assumed to be due to the elution of the originally contained water-soluble low molecular weight components. Table 5 shows the difference in the weight loss between the first day and the seventh day as an index of hydrolyzability. Among these polymers, PGA showed a much higher hydrolyzability of 7.4% than other polymers despite its higher molecular weight. The poly(GBGF) and poly(GPGF) with furandicarboxylic acid units showed low hydrolyzability of 1.3% and 0.8%, respectively. Poly(GBGA) showed slightly higher hydrolyzability (2.0%) than poly(GBGF) and poly(GPGF) but still much lower than PGA. Thus, the poly(GBGF), poly(GPGF), and poly(GBGA) have improved stability in comparison with PGA. PBA and poly(GBGA) have both high biodegradability in seawater and low hydrolyzability. The corresponding random copolymer of poly(butylene adipate-*co*-glycolic acid) with a similar composition (glycolate-content = 60 mol%) was reported to show higher hydrolyzability than PGA [26]. We speculate that the lack of continuous glycolate units in the poly(GBGA) should contribute to its lower hydrolyzability. Thus, the controlled incorporation of glycolate units into copolyesters realized both the suppression of hydrolysis as well as improved biodegradability. 

## 4. Conclusions

Novel sequence-controlled copolyesters composed of glycolic acid, 1,4-butanediol or 1,3-propanediol, and dicarboxylic acids were synthesized from the polycondensation of alkylene diglycolate and the chloride of the corresponding dicarboxylic acid. The *T*_m_ of the poly(GBGT) (202 °C) is comparable to that of poly(GEGT) and higher than that of the poly(GPGT) (121 °C), suggesting an odd–even effect of the diol carbon numbers on their crystallization properties. In contrast to the fact that the corresponding random copolymers are amorphous, the poly(GBGF) showed *T*_m_ at 90 °C, indicating its enhanced crystallinity by its regular sequence. Biodegradation tests in seawater showed that poly(GBGF) and poly(GPGF) have intermediate biodegradability between PGA and PBF. poly(GBGA) showed high seawater-biodegradability, similar to PBA. On the other hand, the hydrolysis of poly(GBGF), poly(GPGF), and poly(GBGA) was slower than that of PGA. Thus, these sequence-controlled copolyesters have both improved biodegradability compared to PBF and lower hydrolyzability than PGA. 

## Figures and Tables

**Table 1:** Synthesis of Poly(GBGT) and Poly(GPGT) ^1^.

Run	Sample	Temp. (°C)	Yield (%)	*M*_n_ (kg mol^−1^)	*M*_w_/*M*_n_
1	Poly(GBGT)	r.t.	86	2.4 ^2^	1.5 ^2^
2	Poly(GBGT)	70	89	1.5 ^2^	2.8 ^2^
3	Poly(GBGT)	100	95	1.0 ^2^	2.9 ^2^
4	Poly(GPGT)	r.t.	64	3.2 ^3^	3.6 ^3^

^1^ Polymerization conditions: time = 2 h, solvent = C_2_H_2_Cl_4_ (TCE), [TC]_0_ = 0.1 M, pyridine (2 mL) was used as a base. ^2^ Determined by GPC calibrated with poly(methyl methacrylate) standards in 1,1,1,3,3,3,-hexafluoro-2-propanol (HFIP). ^3^ Determined by GPC calibrated with polystyrene standards in CHCl_3_.

**Table 2:** Synthesis of Poly(GBGF) and Poly(GPGF) ^1^.

Run	Sample	Temp. (°C)	Yield (%)	*M*_n_^2^ (kg mol^−1^)	*M*_w_/*M*_n_^2^
5	Poly(GBGF)	r.t.	79	10	1.7
6	Poly(GBGF)	50	84	11	1.6
7	Poly(GBGF)	70	92	14	1.6
8	Poly(GBGF)	100	19	2	1.8
9 ^3^	Poly(GPGF)	r.t.	92	4	1.4
10	Poly(GPGF)	r.t.	94	6	1.9
11 ^4^	Poly(GPGF)	-	72	10	1.2

^1^ Polymerization conditions: FC = 0.23—0.33 g, [GXG]_0_: [FC]_0_ = 1: 1.1, time = 2 h, solvent = pyridine (2 mL) was used as a base. ^2^ Determined by GPC calibrated with polystyrene standards in CHCl_3_. ^3^ Polymerization conditions: temp = r.t., time = 2 h, solvent = C_2_H_2_Cl_4_ (TCE), [FC]_0_ = 0.1 M, pyridine (2 mL) was used as a base. ^4^ Obtained by the reprecipitation of the poly(GPGF) sample (run 10) in MeOH.

**Table 3:** Synthesis of Poly(GBGA) ^1^.

Run	Temp. (°C)	Time (h)	Yield (%)	*M*_n_^2^ (kg mol^−1^)	*M*_w_/*M*_n_^2^
11	r.t.	2	70	8	1.5
12	50	2	84	21	1.5
13	50	3.5	92	21	1.5
14	70	2	19	17	1.5

^1^ Polymerization conditions: AC = 0.22—0.27 g (1.07 mmol~1.31 mmol), [GBG]_0_: [AC]_0_ = 1: 1, [GBG]_0_ = 1.0 M, Solvent = C_2_H_2_Cl_4_ (TCE), pyridine (2 mL) was used as a base. ^2^ Determined by GPC calibrated with polystyrene standard in THF.

**Table 4:** Comparison of thermal properties of the sequence-controlled copolyesters having different diol units.

Run	Sample	x	*M*_n_^1^ (kg mol^−1^)	*M*_w_/*M*_n_^1^	*T*_g_^2^ (°C)	*T*_m_^2^ (°C)	Δ*H*_m_^2^ (J/g)
1	Poly(GBGT)	4	2.4	1.5	9	202	28
4	Poly(GPGT)	3	3.2	3.6	33	121	12
-	Poly(GEGT) [13]	2	8.1	2.4	48	209	20
7	Poly(GBGF)	4	14	1.6	34	93	50
11	Poly(GPGF)	3	10	1.2	43	n.d. ^3^	n.d. ^3^
-	Poly(GEGF) [13]	2	17	3.8	68	127	14
12	Poly(GBGA)	4	21	1.5	−31	72	21
-	Poly(GEGA) [13]	2	6.5	1.8	13	67,77	13

^1^ Determined by GPC. ^2^ Determined by DSC. The *T*_m_ and Δ*H*_m_ were read from the first heating scan, while the *T*_g_ was read from the second heating scan. ^3^ Not detected.

**Table 5:** Differences in hydrolysis degree between the first day and the seventh day.

Polymer	Differences in Weight Loss between the First Day and Seventh Day (%)
Poly(GBGF)	1.3
Poly(GPGF)	0.8
PGA	7.4
Poly(GBGA)	2.0
PBA	0.7

## Data Availability

The data presented in this study are available on request from the corresponding author.

## Supplementary Materials

The following supporting information can be downloaded at: https://www.mdpi.com/article/10.3390/polym15051155/s1, Figure S1: 1H NMR spectrum of GPG (CDCl3, 500 MHz, r.t.). Figure S2: 1H NMR spectrum of poly(GPGT) (run 4) (CDCl3, 500 MHz, r.t.) Figure S3: 1H NMR spectrum of poly(GBGF) (Run 5) (CDCl3, 500 MHz, r.t.). Figure S4: 1H NMR spectrum of poly(GPGF) (Run 9) (CDCl3, 500 MHz, r.t.). Figure S5: 1H NMR spectrum of spectrum of poly(GBGA) (Run 14) (CDCl3, 500 MHz, r.t.). Figure S6: DSC profiles of poly(GBGT) (Run 1). Figure S7: DSC profiles of poly(GPGT) (Run 4). Figure S8: DSC profiles of poly(GBGF) (Run 7). Figure S9: DSC profiles of poly(GPGF) (Run 11). Figure S10: DSC profiles of poly(GBGA) (Run 12).
