# Supplementary material for: Synthesis, Properties, and Biodegradability of Novel Sequence-Controlled Copolyesters Composed of Glycolic Acid, Dicarboxylic Acids, and C3 or C4 Diols
Source: Polymers (Basel). 2023 Feb 24;15(5):1155. doi: 10.3390/polym15051155 (PMC10007436; doi:10.3390/polym15051155)
Supplement: Supplementary file 1 [file polymers-15-01155-s001.zip › polymers-2214405-supplementary.pdf]

Supporting Information

Synthesis, properties, and biodegradability of novel se-quence-controlled copolyesters composed of glycolic acid, di-carboxylic acids, and C3 or C4 diols

Yuushou Nakayama[1,*], Keitaro Fukumoto[1], Yuji Kusu[1], Ryo Tanaka[1], Takeshi Shiono[1], Norioki Kawasaki[2], Naoko Yamano[2], Atsuyoshi Nakayama[2]

[1] Department of Applied Chemistry, Graduate School of Advanced Science and Engineering, Hiroshima University, 1-4-1 Kagamiyama, Higashi-Hiroshima Hiroshima 739-8527, Japan.

[2] Biomedical Research Institute, National Institute of Advanced Industrial Science and Technology (AIST), 1-8-31 Midorigaoka, Ikeda, Osaka 563-8577, Japan.

Contents

Figure S1: ^{1}H NMR spectrum of GPG ($CDCl_3$, 500 MHz, r.t.).

Figure S2: ^{1}H NMR spectrum of poly(GPGT) (run 4) ($CDCl_3$, 500 MHz, r.t.).

Figure S3: ^{1}H NMR spectrum of poly(GBGF) (Run 5) ($CDCl_3$, 500 MHz, r.t.).

Figure S4: ^{1}H NMR spectrum of poly(GPGF) (Run 9) ($CDCl_3$, 500 MHz, r.t.).

Figure S5: ^{1}H NMR spectrum of spectrum of poly(GBGA) (Run 14) ($CDCl_3$, 500 MHz, r.t.).

Figure S6: DSC profiles of poly(GBGT) (Run 1).

Figure S7: DSC profiles of poly(GPGT) (Run 4).

Figure S8: DSC profiles of poly(GBGF) (Run 7).

Figure S9: DSC profiles of poly(GPGF) (Run 11).

Figure S10: DSC profiles of poly(GBGA) (Run 12).

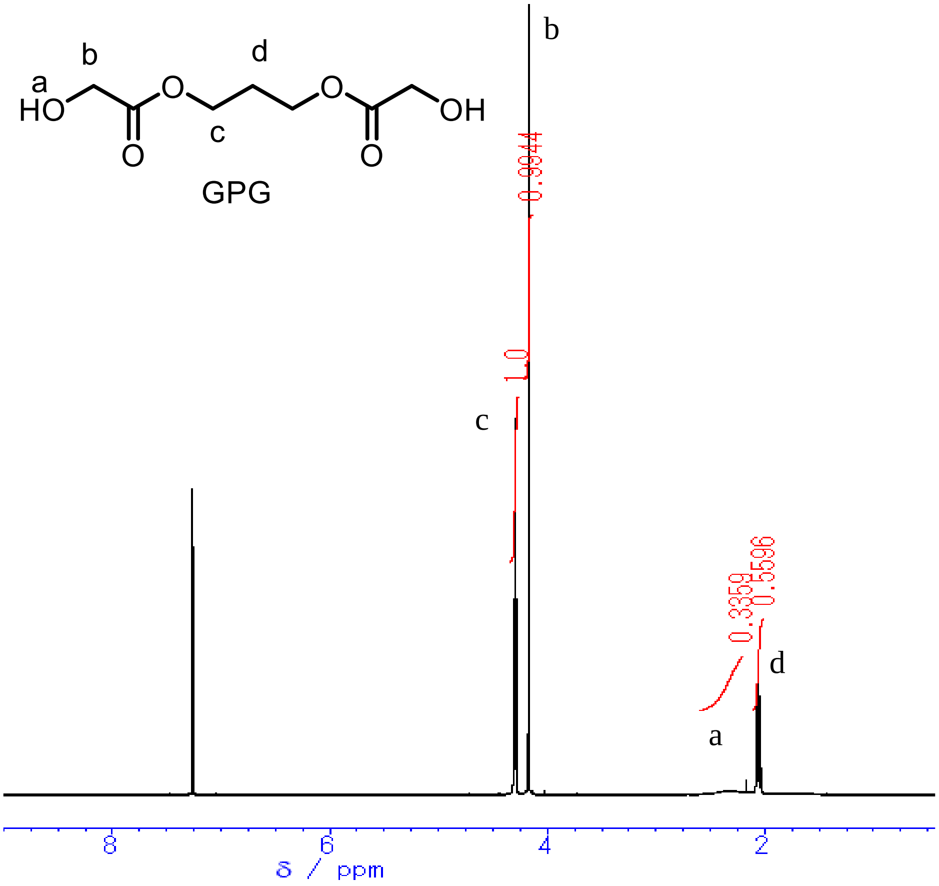

Figure S1: ^{1}H NMR spectrum of GPG ($CDCl_3$, 500 MHz, r.t.).

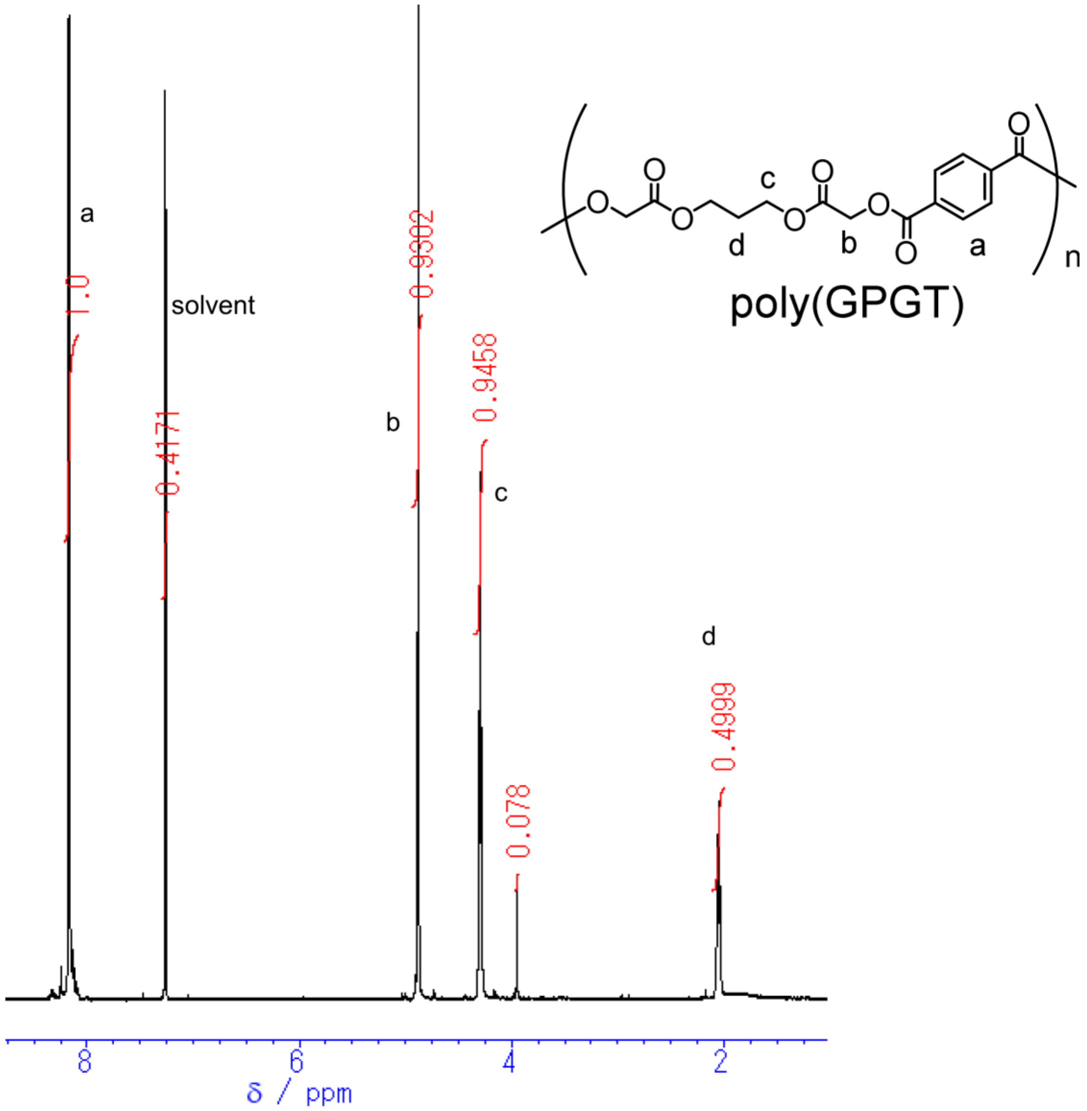

Figure S2: ^{1}H NMR spectrum of poly(GPGT) (run 4) ($CDCl_3$, 500 MHz, r.t.).

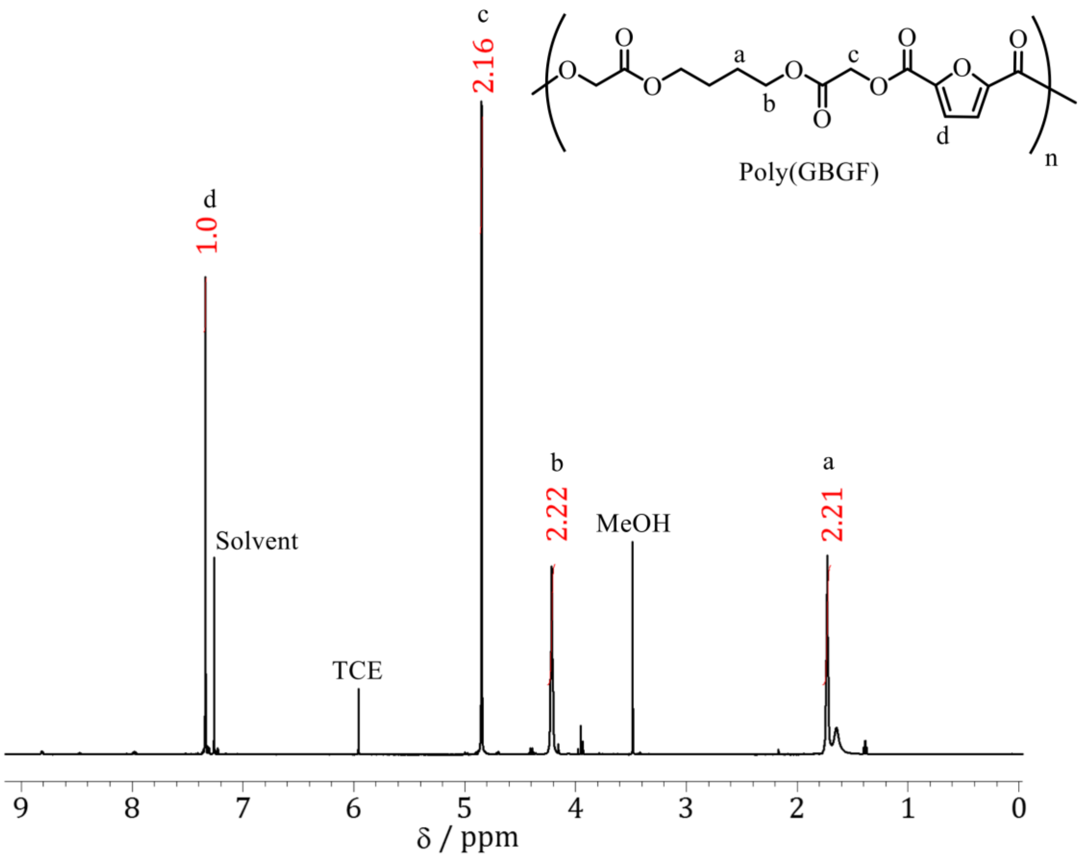

Figure S3: ^{1}H NMR spectrum of poly(GBGF) (Run 5) ($CDCl_3$, 500 MHz, r.t.).

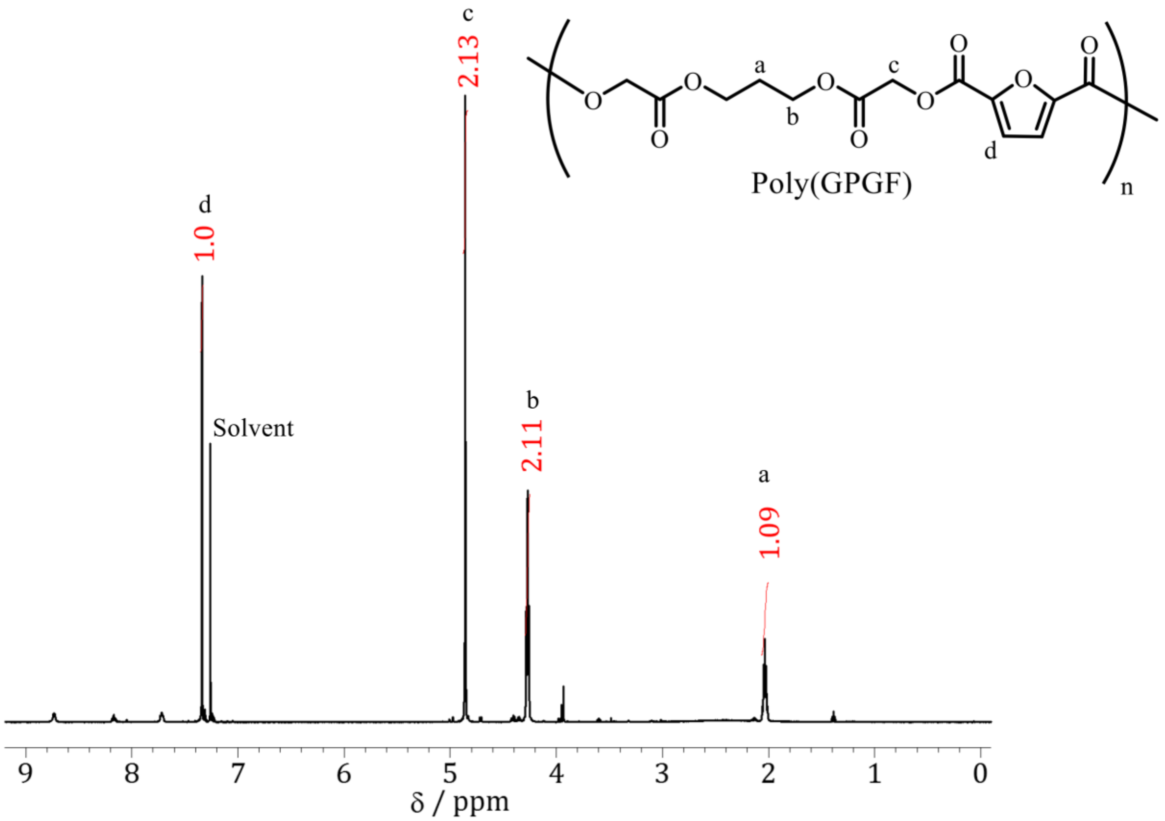

Figure S4: ^{1}H NMR spectrum of poly(GPGF) (Run 9) ($CDCl_3$, 500 MHz, r.t.).

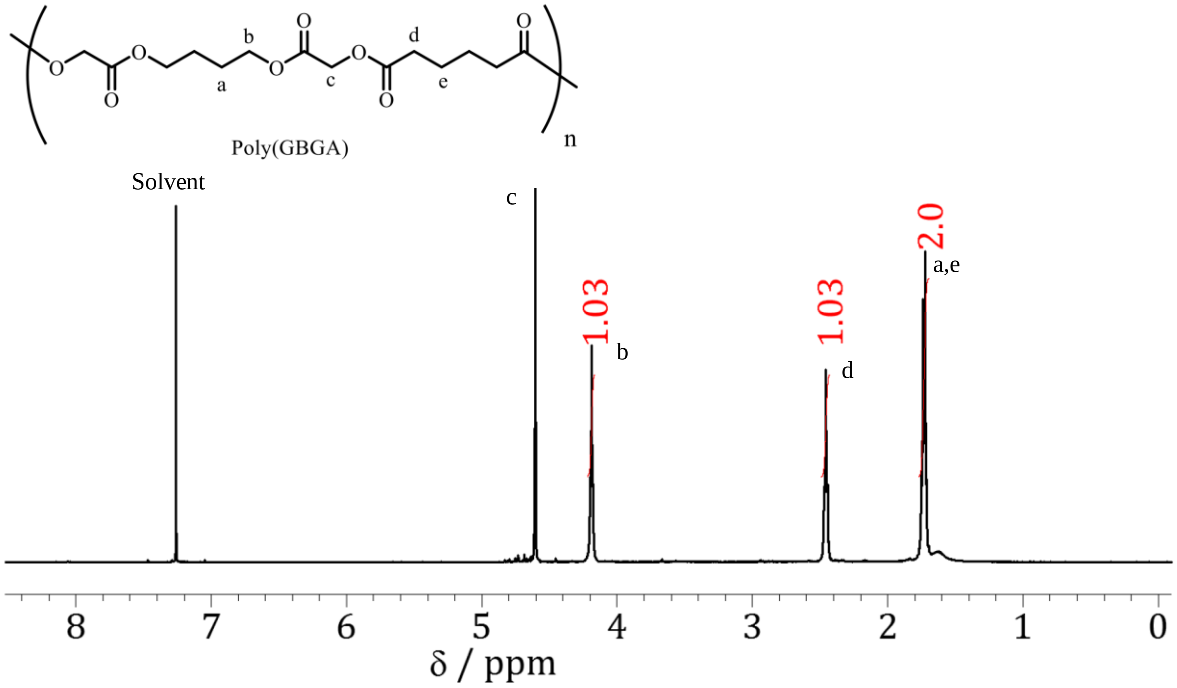

Figure S5: ^{1}H NMR spectrum of poly(GBGA) (Run 14) ($CDCl_3$, 500 MHz, r.t.).

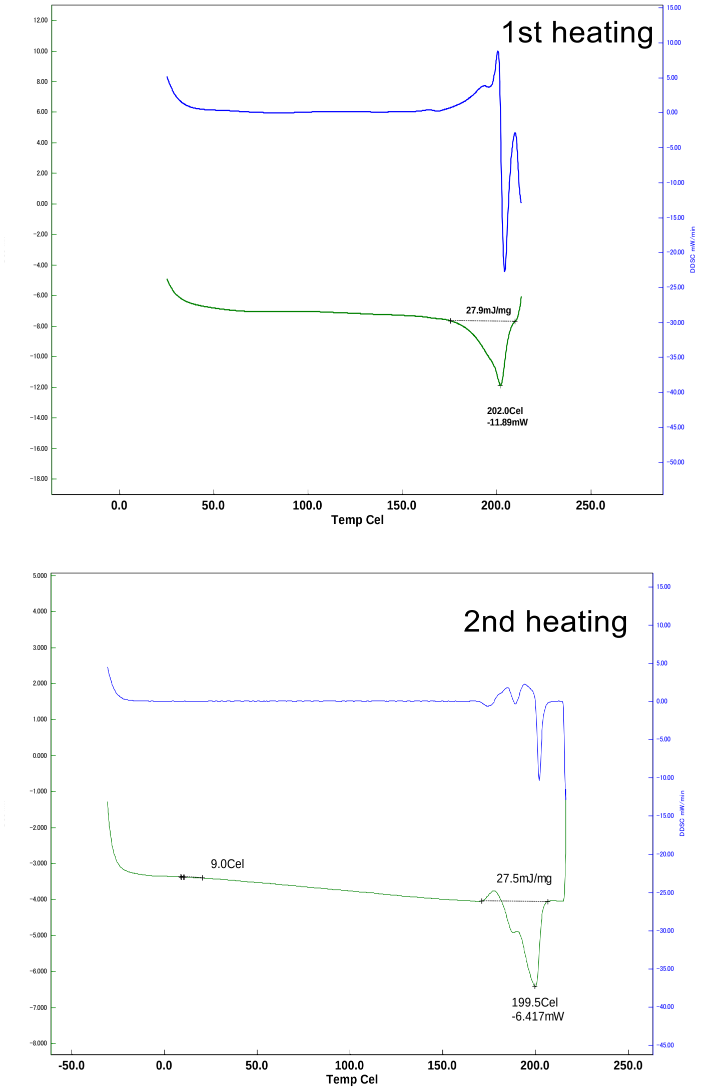

Figure S6: DSC profiles of poly(GBGT) (Run 1).

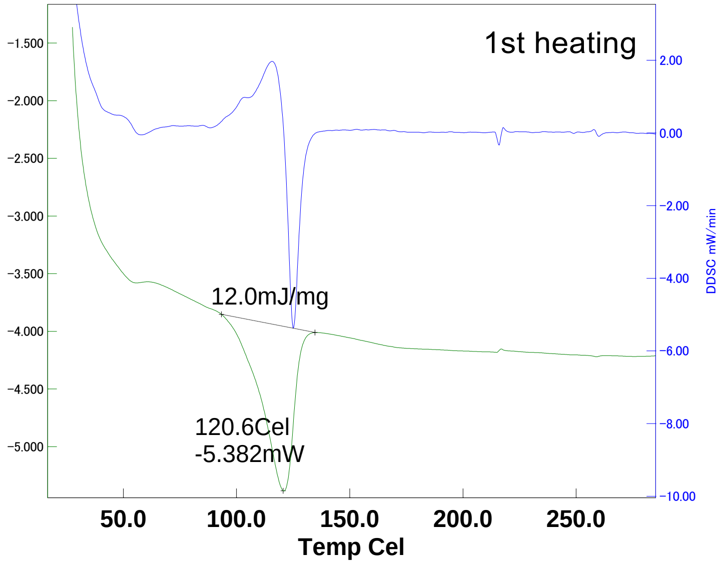

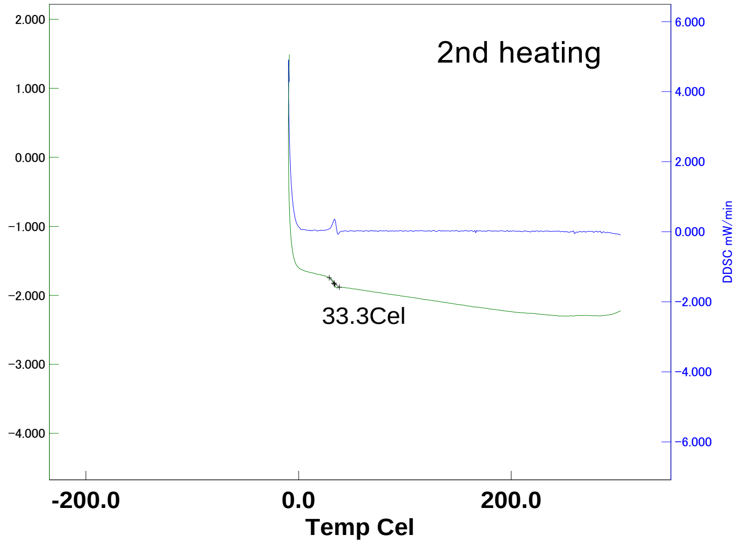

Figure S7: DSC profiles of poly(GPGT) (Run 4).

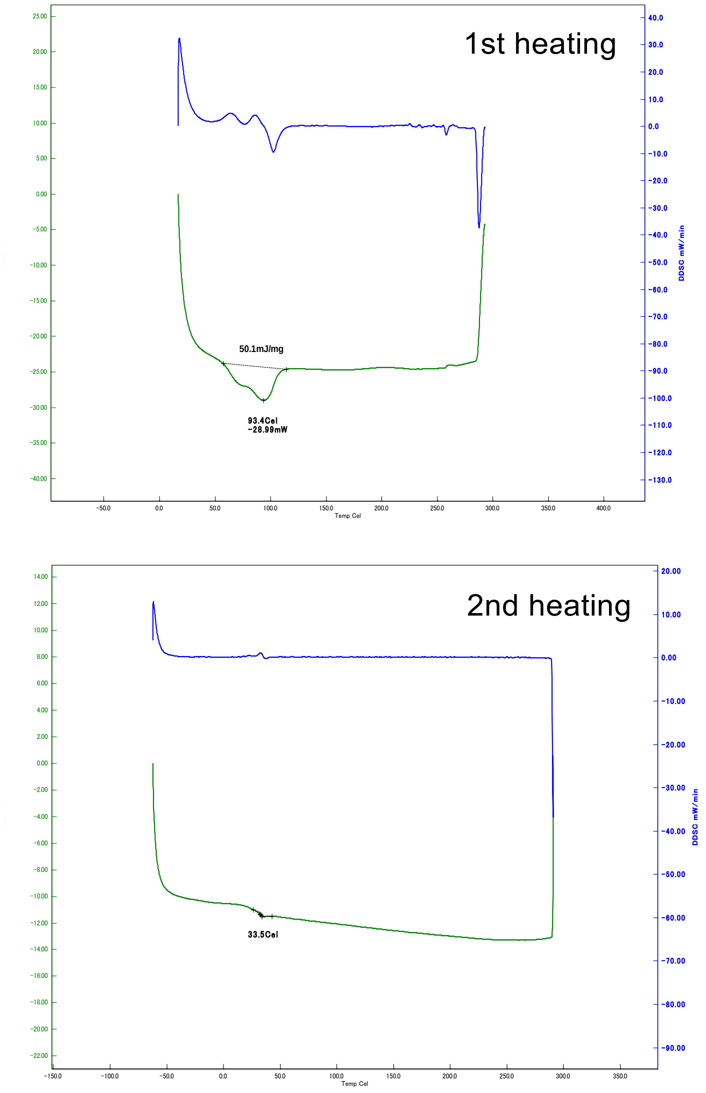

Figure S8: DSC profiles of poly(GBGF) (Run 7).

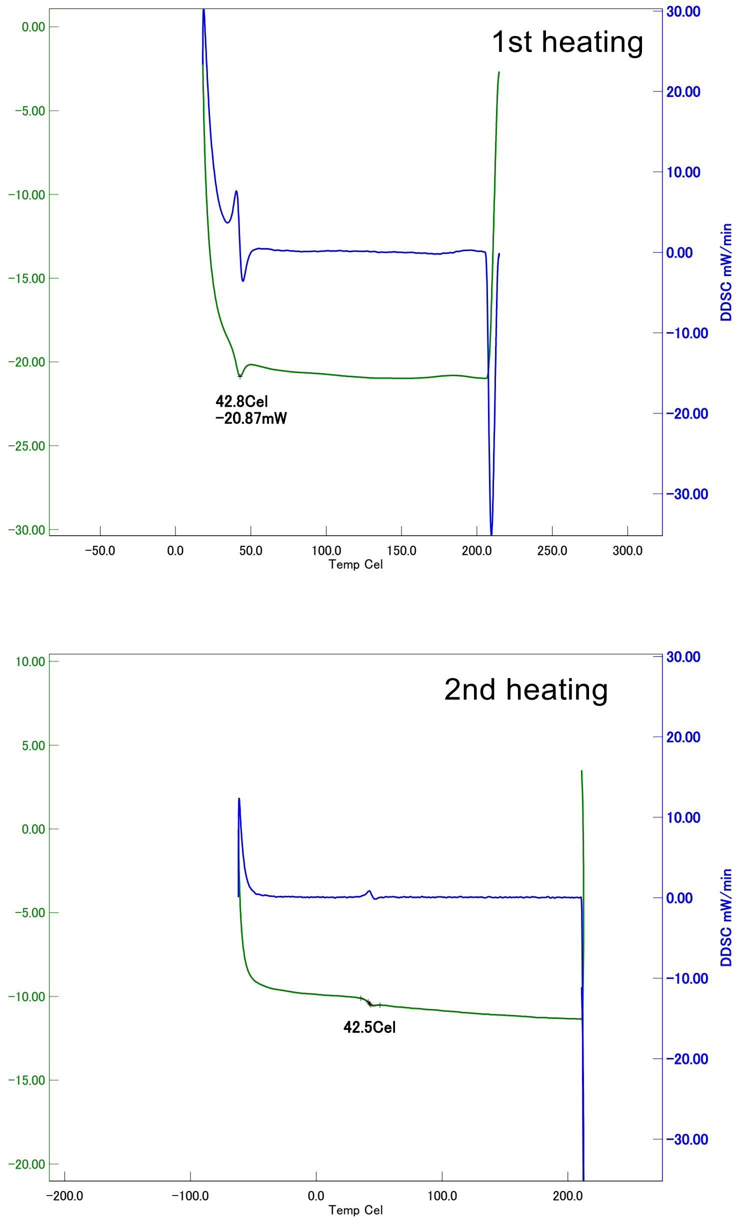

Figure S9: DSC profiles of poly(GPGF) (Run 11).

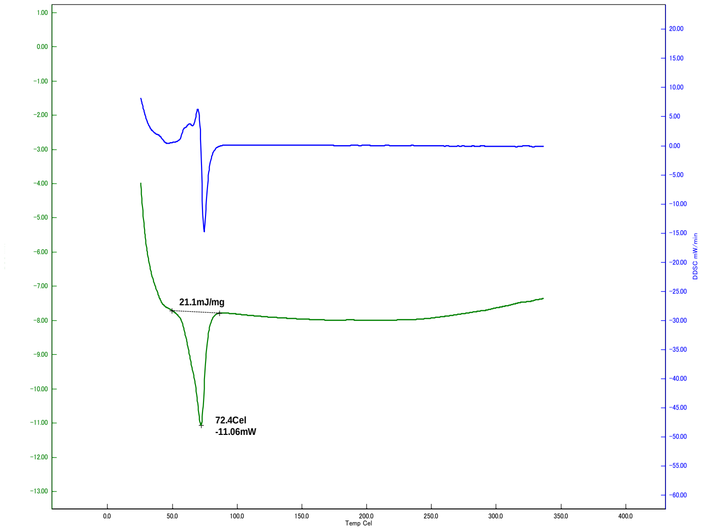

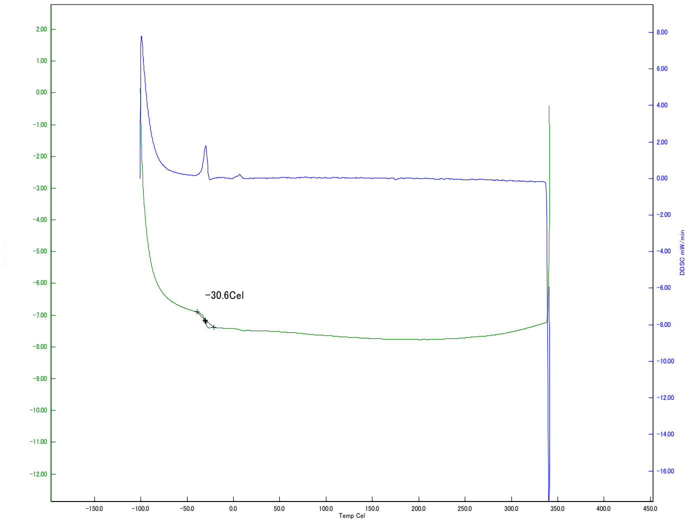

Figure S10: DSC profiles of poly(GBGA) (Run 12).
